# Mechano-Hypoxia Conditioning of Engineered Human Meniscus

**DOI:** 10.3389/fbioe.2021.739438

**Published:** 2021-09-03

**Authors:** Alexander R. A. Szojka, David Xinzheyang Li, Malou E. J. Sopcak, Zhiyao Ma, Melanie Kunze, Aillette Mulet-Sierra, Samer M. Adeeb, Lindsey Westover, Nadr M. Jomha, Adetola B. Adesida

**Affiliations:** ^1^ Department of Surgery, Faculty of Medicine and Dentistry, University of Alberta, Edmonton, AB, Canada; ^2^ Department of Civil and Environmental Engineering, Faculty of Engineering, University of Alberta, Edmonton, AB, Canada; ^3^ Department of Mechanical Engineering, Faculty of Engineering, University of Alberta, Edmonton, AB, Canada

**Keywords:** hypoxia, mechanical loading, dynamic compression, cyclic hydrostatic pressure, human meniscus, tissue engineering, extracellular matrix

## Abstract

Meniscus fibrochondrocytes (MFCs) experience simultaneous hypoxia and mechanical loading in the knee joint. Experimental conditions based on these aspects of the native MFC environment may have promising applications in human meniscus tissue engineering. We hypothesized that *in vitro* “mechano-hypoxia conditioning” with mechanical loading such as dynamic compression (DC) and cyclic hydrostatic pressure (CHP) would enhance development of human meniscus fibrocartilage extracellular matrix *in vitro*. MFCs from inner human meniscus surgical discards were pre-cultured on porous type I collagen scaffolds with TGF-β3 supplementation to form baseline tissues with newly formed matrix that were used in a series of experiments. First, baseline tissues were treated with DC or CHP under hypoxia (HYP, 3% O_2_) for 5 days. DC was the more effective load regime in inducing gene expression changes, and combined HYP/DC enhanced gene expression of fibrocartilage precursors. The individual treatments of DC and HYP regulated thousands of genes, such as chondrogenic markers *SOX5/6*, in an overwhelmingly additive rather than synergistic manner. Similar baseline tissues were then treated with a short course of DC (5 vs 60 min, 10–20% vs 30–40% strain) with different pre-culture duration (3 vs 6 weeks). The longer course of loading (60 min) had diminishing returns in regulating mechano-sensitive and inflammatory genes such as *c-FOS* and *PTGS2*, suggesting that as few as 5 min of DC was adequate. There was a dose-effect in gene regulation by higher DC strains, whereas outcomes were inconsistent for different MFC donors in pre-culture durations. A final set of baseline tissues was then cultured for 3 weeks with mechano-hypoxia conditioning to assess mechanical and protein-level outcomes. There were 1.8–5.1-fold gains in the dynamic modulus relative to baseline in HYP/DC, but matrix outcomes were equal or inferior to static controls. Long-term mechano-hypoxia conditioning was effective in suppressing hypertrophic markers (e.g., *COL10A1* 10-fold suppression vs static/normoxia). Taken together, these results indicate that appropriately applied mechano-hypoxia conditioning can support meniscus fibrocartilage development *in vitro* and may be useful as a strategy for developing non-hypertrophic articular cartilage using mesenchymal stem cells.

## Introduction

The knee menisci are fibrocartilaginous structures that serve to protect the articular cartilage from excessive stress. Their functional extracellular matrix (ECM) is mainly composed of type I collagen (Fox et al., 2012; Makris et al., 2011). The inner meniscus regions contain lesser amounts of hyaline cartilage-type matrix components such as type II collagen and aggrecan (Fox et al., 2012; Makris et al., 2011). Meniscus ECM is synthesized by cells known as meniscus fibrochondrocytes (MFCs) (Sanchez-Adams and Athanasiou, 2009). In adults, the inner regions are avascular and unable to heal upon injury (Arnoczky and Warren, 1982). These injuries are associated with early osteoarthritis (OA) development (Lohmander et al., 2007). The objective of meniscus tissue engineering is to generate cellular replacements that prevent OA development upon implantation.

Hypoxia (HYP) and mechanical loading are characteristic features of the native MFC environment that influence development of the avascular fibrocartilage phenotype in the inner meniscus regions (Fox et al., 2012; Makris et al., 2011; Lund-Olesen, 1970). For example, the meniscus exists in synovial fluid with oxygen saturation ranging from 1–7% and experiences axial compressive strains of ∼5–10% under body weight (Lund-Olesen, 1970; Freutel et al., 2014). Individual HYP and mechanical loading [e.g., dynamic compression (DC), cyclic hydrostatic pressure (CHP)] treatments and their combination (“mechano-hypoxia conditioning”) can be useful tools for engineering cartilaginous tissues in cells including MFCs and mesenchymal stem cells (MSCs) through the molecular actions of factors including hypoxia-inducible factor-1 (HIF-1), SRY-Box transcription factor 9 (SOX9), and transforming growth factor-beta (TGF-β) (Mauck et al., 2000; AufderHeide and Athanasiou, 2004; Adesida et al., 2006; Suzuki et al., 2006; Baker et al., 2010; Egli et al., 2011; Huey and Athanasiou, 2011; Tan et al., 2011; Furumatsu et al., 2012; Kanazawa et al., 2012; Guilak et al., 2014; McNulty and Guilak, 2015; Zellner et al., 2015; Anderson and Johnstone, 2017; Salinas et al., 2018; Szojka et al., 2019). Hypoxia and mechanical loading do not always have beneficial effects with regards to modulation of ECM components in engineered meniscus (e.g., types I and II collagen, aggrecan), however, suggesting the need to identify appropriate culture parameters (Szojka et al., 2021a). In human engineered meniscus, a short-term (1-day) mechano-hypoxia treatment (30–40% dynamic compressive strain, 3% O_2_) promoted fibrocartilage gene expression (Szojka et al., 2021b) after a static pre-culture period, but the effects of long-term treatments and different loading types and parameters have not been explored (Szojka et al., 2021b). Our overarching hypotheses were that longer mechano-hypoxia treatments would also have pro-fibrocartilage effects and that they would translate into beneficial outcomes at mechanical and protein levels.

Thus, we first compared mechano-hypoxia treatments (DC and CHP) and characterized the transcriptome response to a longer (5 days) course of treatment than before (Szojka et al., 2021b). We then manipulated loading incident duration, applied strain, and pre-culture duration to better understand how they affect gene expression. Using this information, we finally investigated the long-term (3 weeks) outcomes of mechano-hypoxia conditioning after a pre-culture period for initial matrix formation.

## Materials and Methods

**FIGURE 1:**
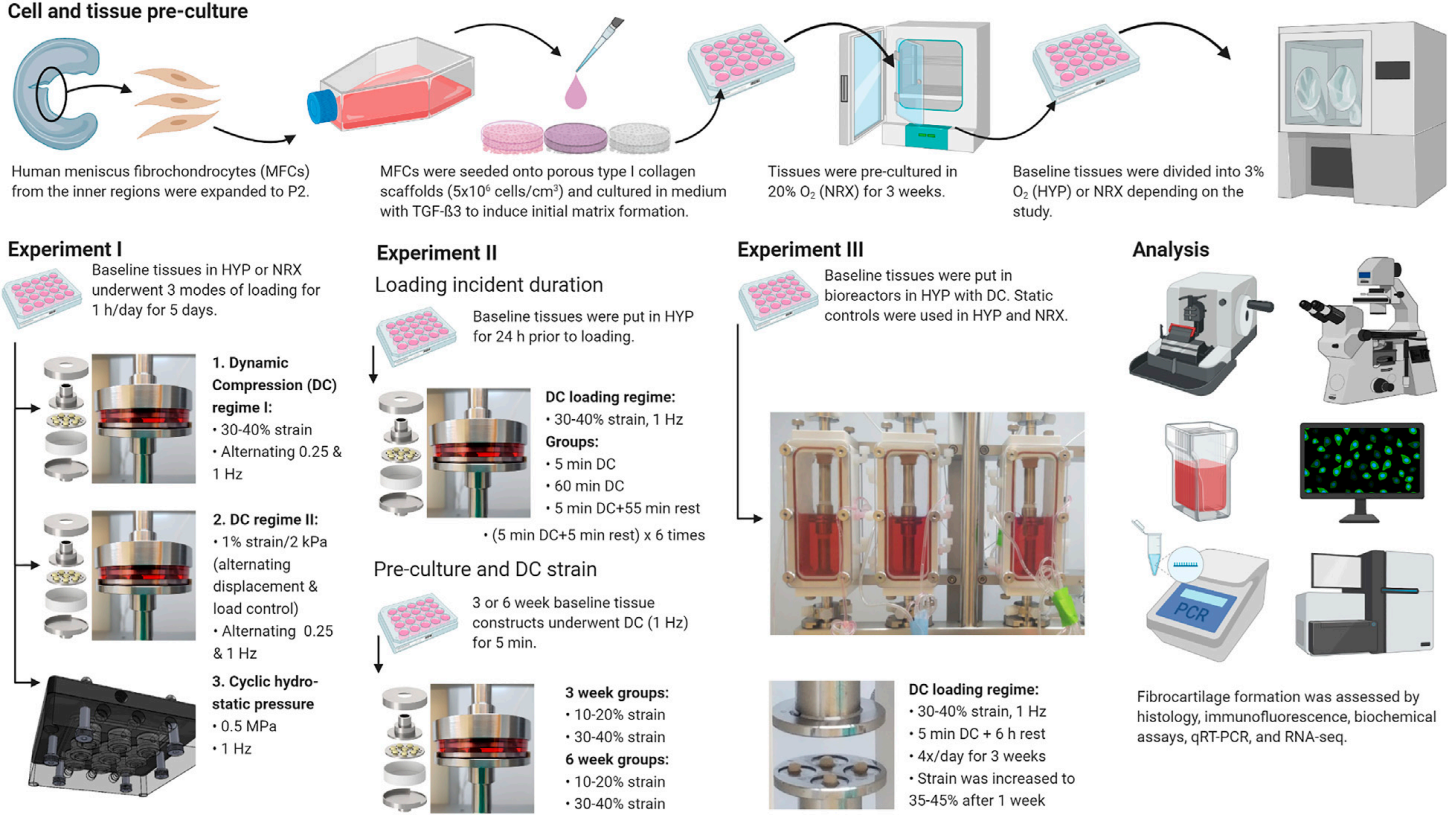
Experiment outlines. Created with Biorender.com (2021).

Most culture methods and assays were performed identically to those described in previous work (Adesida et al., 2012; Liang et al., 2017). The experiments are outlined in Figure 1. Experimental parameters in each experiment were selected based upon previous experiences and new data as it became available, including from pilot studies of scaffold seeding density, orbital shaking, and number of loading days (Supplementary Material) (Bornes et al., 2016; Szojka et al., 2021a; Szojka et al., 2021b; Szojka et al., 2021c).

### Ethics Statement and Sample Collection

Human inner meniscus tissues (*n* = 6 donors) were collected during arthroscopic partial meniscectomies with approval from the University of Alberta Health Research Ethics Board—Biomedical Panel (#Pro00018778). Non-identifying donor details and their allocation to each experiment are presented in the Supplementary Material Tables file.

### Cell and Tissue Pre-Culture

Meniscus fibrochondrocytes (MFCs) were isolated by collagenase-mediated digestion for 22 h (Type II, 0.15% w/v, Worthington, NJ, United States, #LS004176) and expanded in high-glucose (4.5 g/L) Dulbecco’s Modified Eagle Medium (DMEM) (Sigma-Aldrich, United States, #D6429) with 10% fetal bovine serum (FBS, Sigma, United States, #F1051-500ml) and combined TGF-β1 (1 ng/ml, ProSpec, Israel, #cyt-716c) and basic fibroblast growth factor (FGF-2, 5 ng/ml, ORF Genetics, Iceland, 01-A01110). They were stored in liquid nitrogen after passage 1 and then further expanded to passage 2 (6.5 ± 0.9 total population doublings).

MFCs were seeded onto bovine type I collagen scaffolds (diameter = 6 mm, height = 3.5 mm, pore size = 115 ± 20 μm, Integra LifeSciences, NJ, United States) at a density of 5×10^6^ cells/cm^3^ (Adesida et al., 2012; Bornes et al., 2016). No measurable glycosaminoglycan (GAG) and DNA contents were reported on these scaffolds, and compressive modulus was less than 2 kPa (Szojka et al., 2021c). Cell-seeded scaffolds were cultured for 3 weeks for baseline matrix formation using a standard serum-free chondrogenic medium with 10 ng/ml TGF-β3 (Proteintech Group, United States, #HZ-1090) and normoxia (NRX) incubators (∼20% O_2_, 5% CO_2_). Tissues were cultured on a gentle orbital shaker in experiment I only (Supplementary Material).

### Manipulated Variables

After baseline matrix formation, tissue replicates were randomly assigned into conditions and transferred into an X3 incubator system to apply loading with continuous gas control (Biospherix, United States). Dynamic compression (DC) and cyclic hydrostatic pressure (CHP) loading were applied, respectively, using a Biodynamic 5210 system (TA Instruments, United States) and a MechanoCulture TR (CellScale, Canada). In experiments I ane II, tissues were housed in a previously-described custom bioreactor chamber that is optimal for short-term loading events (Szojka et al., 2021b). In the longer-term experiment III, tissues were housed in commercial bioreactor chambers that permit automated loading events, i.e., with no user input apart from initial setup, to facilitate long-term experimentation. In experiments I and II, tissues were harvested 30 min after the end of each loading period, whereas in experiment III they were harvested 6 h after due to the time required to dismantle the bioreactors. Gene expression samples were stored in TRIzol reagent for later analysis (ThermoFisher Scientific, United States, reference #15596018) at −80°C.

#### Experiment I

Tissues were treated for 5 days in continuous hypoxia (HYP) or NRX (3 or 20% O_2_). Tissues were loaded with either DC (regime I: 30–40% sinusoidal strain; regime II: 1% compression in displacement control then off-load in load control to 2 kPa to maintain platen contact with tissues) with frequency alternating 0.25 and 1 Hz cycle-to-cycle, or CHP from 0–0.5 MPa (the maximum capability of the device) at 1 Hz for 1 h daily. The static strain offset in DC regime I (30%) was based upon previous work with type I collagen scaffolds and may not be unreasonable for free-swelling engineered meniscus tissues, given that free-swelling samples of native meniscus swelled by an average of 18% (Andrews et al., 2015; Scholtes et al., 2018; Szojka et al., 2021b). The 1% strain/cycle parameter in DC regime II was based upon a test run that found 2% deformation per cycle led to much more strain accumulation (>60%) than desired, and the 2 kPa stress during decompression was the smallest value the actuator could reliably be controlled to without unstable and noisy motion. For DC static vehicle controls (DC VCtrl), tissues were brought into contact with the platens without compression. For CHP static controls (CHP VCtrl), tissues were sealed in the loading device under the appropriate oxygen level but were not loaded. There were four replicates for gene expression and 1 for histological analysis in all conditions for all donors (2 oxygen tensions × 5 loading groups × 5 replicates × 3 donors = 150 total tissues).

#### Experiment II

Baseline tissues were transferred into HYP 24 h prior to DC loading (30–40% strain, 1 Hz) in the following groups: i. VCtrl (as before in experiment I), ii. 5c (5 min), iii. 60c (60 min), iv. 5c+55r (5 min of DC, 55 min of rest), v. (5c + 5r)×6 (cyclic rest, 5 min of DC and 5 min of rest repeated six times) (Egli et al., 2011). There were four replicates in all conditions for gene expression (5 conditions × 4 replicates × 3 donors = 60 total tissues).

In the second part of experiment II, tissues were maintained in NRX for DC loading (5 min, 1 Hz). The baselines were pre-cultured for 3 or 6 weeks and were then divided into three DC loading groups: VCtrl (as before), 10–20% strain, 30–40% strain. There were shortages of cells and thus not all the three donors could be represented in each condition and there were only 2-4 replicates per condition for gene expression (42 total tissues).

#### Experiment III

Baseline tissues (3-weeks pre-culture) were randomly divided into three groups: NRX/static, HYP/static, and HYP/DC with three donors cultured and loaded with DC simultaneously. Static groups were free swelling rather than true vehicle controls as in experiments I/II due to a shortage of available bioreactors. Medium was circulated from the bioreactors into a hypoxic incubator chamber for gas exchange using a peristaltic pump. Based on outcomes from experiments I/II, DC loading was applied four times a day from 30–40% strain at 1 Hz for 5 min per loading incident for 3 weeks. After 1 week, strain levels were increased to 35–45% to compensate for some permanent compaction. There were four replicates per condition (the maximum capacity of the bioreactor platens), which were cut in half (eight total halves) and allocated to gene expression (three halves), glycosaminoglycan (GAG) and DNA (three halves), and histology (two halves) (3 conditions × 4 replicates × 3 donors = 36 tissues in total).

### Outcome Variables

#### RNA Extraction, Quantitative Real-Time Polymerase Chain Reaction, and RNA-Seq

RNA was isolated with isopropanol precipitation or PureSPIN MINI spin columns (Luna Nanotech, Canada, #NK051-250). For experiment I, equal masses of RNA from replicates within conditions and donors was combined into pools to reduce nuisance inter-replicate variability. RNA was reverse transcribed into cDNA and amplified by quantitative real-time polymerase chain reaction (qRT-PCR) using gene specific primers listed in the Supplementary Material Tables file.

Data was presented using the 2^ΔΔCt^ method using the mean expression level of reference genes *β-actin*, *B2M*, and *YHWAZ* and reference groups for fold changes as indicated in figure captions (Livak and Schmittgen, 2001). Statistical analysis was performed in SPSS 27 (IBM, United States) using ΔCt values. Analysis of variance (ANOVA) was performed with treatment groups as fixed factors and donor as a random factor followed by Tukey’s post hoc test in experiments II/III. No *p* value corrections were applied unless indicated (i.e., in the RNA-seq dataset for q values). Further, differences were considered as significant if *p* < 0.05 unless otherwise indicated. The Shapiro-Wilk test was used to assess approximate normality. Supplementary data files are available (Supplementary Material).

In experiment I, the RNA pools from DC 30–40% groups were sequenced at the University of British Columbia Biomedical Research Centre using Illumina NextSeq 500 with 20 million paired end 42 bp × 42 bp reads. Partek® Flow® software was used for analysis (Version 10.0.21.0302, Copyright © 2021, Partek Inc., St. Louis, MO, United States). Alignment to the human genome hg38 was performed using STAR-2.7.3a and genes were quantified using the Partek E/M algorithm to an annotation model (hg38–RefSeq Transcripts 94; 2020-05-11). A noise reduction filter was applied, and gene counts were normalized using the Add: 1.0, TMM, and Log 2.0 methods. Differential expression analysis was conducted using analysis of variance (ANOVA) for oxygen tension and mechanical loading with donor as a random factor. Significant differences in gene expression were assessed using a combination of minimum total gene counts, unadjusted *p*-values, adjusted *p*-values for false discovery rate (FDR) (q-values), and minimum absolute fold-changes. Principal component analysis (PCA), heatmaps, and visualization of differentially expressed genes (DEGs) were all conducted in Partek Flow software. MCL (Markov Cluster Algorithm) was used to cluster genes in the graph convolution network (GCN) by the structure of the node-edge graph. The raw RNA-seq datasets are deposited in the NCBI GEO database (accession: GSE180467).

#### Histology and Glycosaminoglycan/DNA

Tissues were fixed in formalin overnight at 4°C, paraffin-embedded, sectioned at 5 μm, and stained for Safranin O (Sigma-Aldrich, United States, #S2255-25G), Fast Green FGF (Sigma-Aldrich, United States, #F7258-25G), and Haematoxylin (Sigma-Aldrich, United States, #MHS32-1L) or else labelled with primary antibodies against human types I and II collagens (rabbit anti-human type I collagen, Cedarlane, Canada, #CL50111AP-1; mouse anti-human type II collagen, #II-II6B3, Developmental Studies Hybridoma Bank, United States) for immunofluorescent visualization by secondary antibodies (goat anti-rabbit, #ab150080, Abcam, United Kingdom; goat anti-mouse, #ab150117, Abcam, United Kingdom). There was no detected cross-reaction of the human anti-type I collagen antibody to the bovine type I collagen scaffold. For GAG and DNA, tissues were digested overnight in proteinase K (Sigma-Aldrich, United States, #P2308). GAG was measured by the dimethylmethylene blue (DMMB, Sigma-Aldrich, United States, #341088) assay with a chondroitin sulfate standard (Sigma-Aldrich, United States, #C8529) and DNA by the CyQUANT Cell Proliferation Assay (ThermoFisher Scientific, United States, #C7026).

## Results

### Experiment I: 5-days Mechano-hypoxia

**FIGURE 2:**
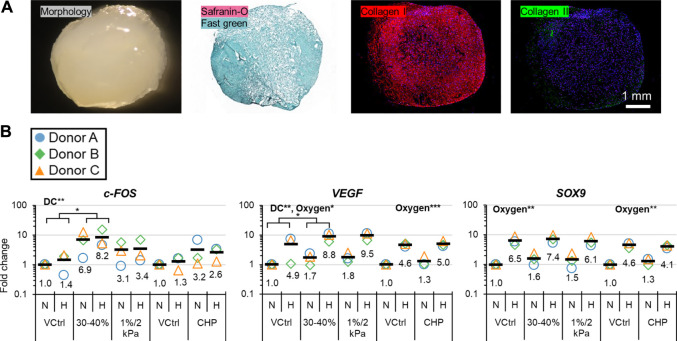
Experiment I: **(A)** Representative baseline tissue matrix morphology (3 weeks preculture), and **(B)** gene expression in response to 5-days of mechano-hypoxia treatment. DC and CHP each had their own VCtrl group since the loading occurred in different chambers. Fold changes are with respect to the appropriate N/VCtrl group within each donor, and values for each gene of interest are normalized to the mean expression of housekeeping genes *β-actin*, *B2M*, and *YWHAZ*. Expression was measured 30 min after the final loading event. Two-way ANOVA was performed twice, once for DC and once for CHP groups. “DC” and “Oxygen” indicate that these factors had a significant main effect. *: *p* < 0.05, **: *p* < 0.01. CHP, Cyclic hydrostatic pressure; DC, dynamic compression; H, hypoxia/HYP; N, normoxia/NRX; VCtrl, static vehicle control.

Baseline tissues (3-weeks pre-culture) were mainly fibrous with minimal staining for hyaline cartilage-type matrix (Figure 2A). Strain accumulated to 40–45% each day in dynamic compression (DC) 1%/2 kPa with peak stresses being lower than in DC 30–40% (Supplementary Figure S4.1); this indicated that DC 1%/2 kPa was the less aggressive DC loading regime. *c-FOS* and *VEGF* were measured as loading and hypoxia (HYP)-sensitive genes, respectively, and *SOX9* as an indicator for hyaline cartilage-type matrix expression. DC 30–40% upregulated *c-FOS* and *VEGF* compared to the static vehicle controls. However, there were no significant differences in gene regulation for DC 1%/2 kPa and cyclic hydrostatic pressure (CHP) (Figure 2B). HYP (5 days) promoted *VEGF* and *SOX9* compared to normoxia (NRX) and to a greater extent than the 1-day HYP treatment previously (Figure 2B) (Lohmander et al., 2007). DC 30–40% was deemed the most effective loading regime and thus it proceeded to RNA sequencing.

#### Experiment I: 5-days Mechano-Hypoxia Transcriptomes

**FIGURE 3:**
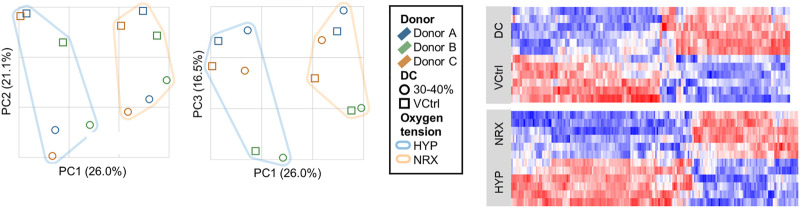
Experiment I: Principal component analysis and heatmaps of the RNA-seq dataset from the 5-days mechano-hypoxia treatment. Expression was measured 30 min after the final loading event. DC, dynamic compression; HYP, hypoxia; NRX, normoxia; PC, principal component; VCtrl, static vehicle control.

Principal components (PCs) 1–3 explained 63.5% of variability and showed clustering for oxygen tension, DC, and donor, This indicated that each variable had generally consistent effects (Figure 3).

**FIGURE 4:**
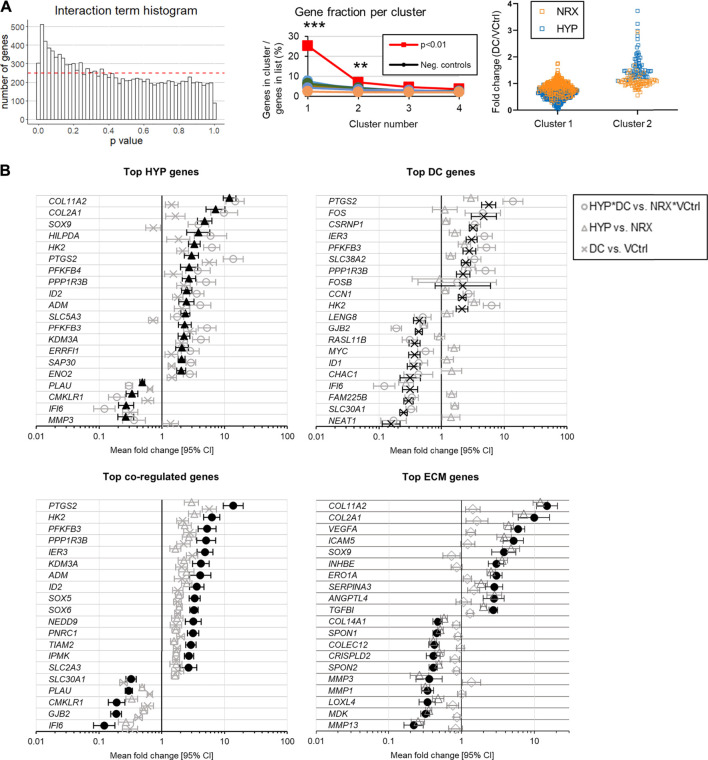
Experiment I: **(A)** Interaction analysis after the 5-days mechano-hypoxia treatment. The dashed red line indicates the expected bin height for a uniform distribution. **(B)** Genes with the largest absolute fold changes for each group (q < 0.05, total count >250). Expression was measured 30 min after the final loading event. CI: confidence interval. Neg, negative.

Most genes were regulated independently, i.e., by only one treatment (Table 1). A smaller set of genes were coregulated, i.e., with significant effects for both hypoxia and DC (Table 1). There were few genes detected with significant DC*HYP interactions, i.e., where gene regulation by one variable depended on the level of the other (Table 1). The interaction *p* value distribution skewed right, indicating that there were true interactions, but that detection power was limiting for individual genes (Figure 4A, left). It was inferred that a biologically relevant interaction would have affected groups of related genes, resulting in regulation profiles that could be detected by cluster analysis. Two clusters were identified among the 328 genes with interaction *p* < 0.01 (selected as a more conservative threshold for this analysis) that contained a significantly larger fraction of the 328 genes than observed in ten negative control lists of equal size with interaction *p* values descending from *p* = 1 based on one sample t tests (Figure 4A, centre). This indicated that these genes were likely being regulated together due to a true interaction. Within the first cluster, genes tended to be downregulated by DC and the effect was more prominent in hypoxia, whereas in the second cluster genes tended to be upregulated by DC and the effect was more prominent in hypoxia (Figure 4A, right). The lists of genes in these clusters are provided in the Supplementary Material. Of the four genes with significant (q < 0.05) interactions, three appeared in cluster 1 (Supplementary Figure S4.2).

**TABLE 1:** Number of genes for each factor meeting combinations of filters.

Filter applied			Genes in group			
Probabilistic	Count	|FC|	DC	HYP	Coregulation	Interaction
*p* < 0.05	None	None	5,392	6,181	2,660	1,288
*p* < 0.05	>250	None	2,804	3,413	1,516	692
q < 0.05	None	None	4,048	5,145	1,682	4
q < 0.05	>250	None	2,128	2,915	1,001	4
q < 0.05	>250	>1.5	122	377	29	NA
q < 0.05	>250	>2	29	104	7	NA

Coregulation indicates significance for both DC and HYP factors.

The top HYP-regulated genes were more clearly related to fibrocartilage matrix (e.g., *COL2A1*, *SOX9*) than the top DC-regulated genes, which instead tended to be nucleus or inflammation related (e.g., *c-FOS*, *PTGS2*) (Figure 4B). Gene Ontology (GO) terms related to the extracellular matrix (ECM) were then used to generate a gene list to more directly assess how each treatment affected fibrocartilage matrix expression (Figure 4, bottom right). Both treatments but especially HYP promoted a favourable expression profile in these genes (Figure 4B, bottom right). GO analysis for the interaction clusters, individual treatments, and coregulation genes are provided in Supplementary Figure S4.3. There were no differences between groups in type I or II collagen immunofluorescence, indicating the gene expression effects had not yet resulted in detectable differences between groups in fibrocartilage matrix (Supplementary Figure S4.4A).

### Experiment II: Loading Incident Duration, Strain and Pre-Culture Duration Parameters

#### Loading Incident Duration

**FIGURE 5:**
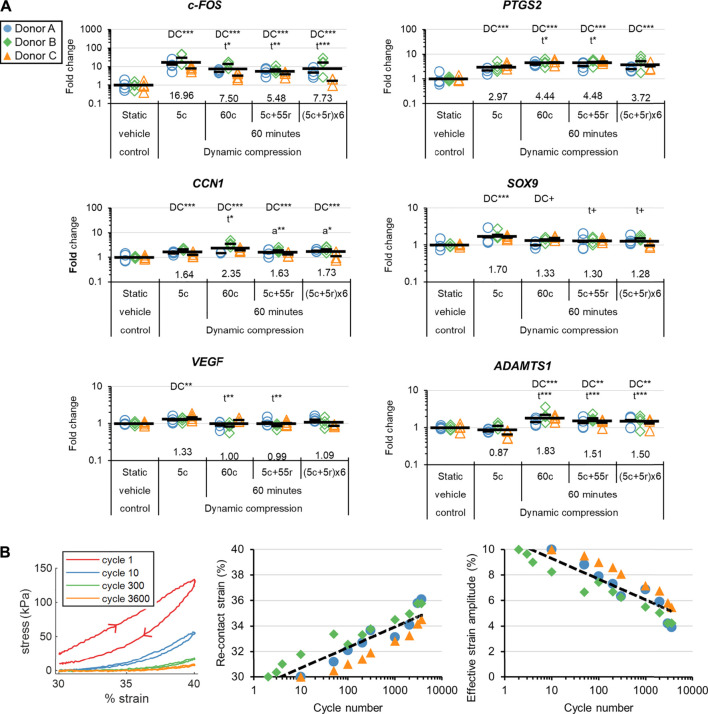
Experiment II: **(A)** The gene expression effects of DC loading incident duration. Fold changes are with respect to the static vehicle control group within each donor, and values for each gene of interest are normalized to the mean expression of housekeeping genes *β-actin*, *B2M*, and *YWHAZ*. Expression was measured 30 min after the loading event. “DC” indicates that a group is significantly different than the static vehicle control. “t” indicates that a DC group is significantly different than 5c. “a” indicates that a 60-min group is significantly different than 60c. ***: *p* < 0.001, **: *p* < 0.01, *: *p* < 0.05, +: *p* < 0.10. **(B)** Loading analysis for the 60c group. The arrowheads show the direction of onloading and offloading. The re-contact strains were defined as the points within the compression phase when the force rose above 0 N, indicating contact. The effective strain amplitudes are calculated as 40% minus the strain at the re-contact points.

Relaxation reached 50, 90, and 95% completion after 14 ± 4, 336 ± 109, and 814 ± 161 cycles based on the 60c group (Supplementary Figure S4.5). The stress vs time curves clearly showed there was an early “viscous, fast relaxation” zone and a late “elastic, slow-relaxation” zone (Supplementary Figure S4.5). Accordingly, gene expression differences induced by DC quickly saturated and reached similar outcomes after accounting for the time lag in harvest, i.e., by comparing the 60c and 5c + 55r groups (Figure 5A and Supplementary Figure S4.6). The selection of measured genes was related to the early response to stimuli, inflammation, the hyaline cartilage phenotype, angiogenesis, and matrix degradation. DC loading in the viscous, fast-relaxation zone was clearly most important for regulation of these genes. The stress began to fall to 0 kPa within the decompression phase of a single cycle after just 50 cycles for all donors, indicating that the tissues were not fully rebounding (Figure 5B). The effective strain amplitude, i.e., the actual strain amplitude experienced by the tissues while in contact with the platen, fell to about 5% by the end of the loading period, helping to explain why continued cycling beyond 5 min had marginal effects on gene expression (Figure 5B).

#### Strain and Pre-Culture Duration

**FIGURE 6:**
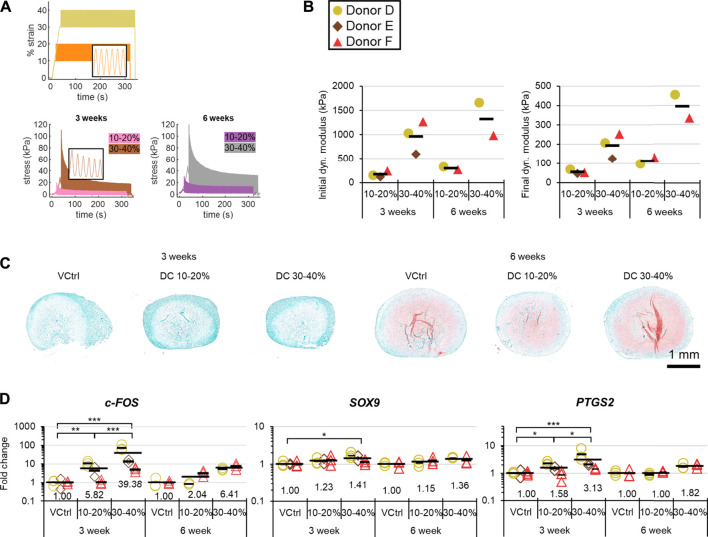
Experiment II: The matrix and gene expression effects of pre-culture duration and strain. **(A)** Representative strain and stress vs time curves. **(B)** Mechanical analysis. **(C)** Representative safranin-O staining after loading in each group. **(D)** Gene expression post-DC loading by qRT-PCR. Fold changes are respect to the VCtrl group within each time point within each donor, and values for each gene of interest are normalized to the mean expression of housekeeping genes *β-actin*, *B2M*, and *YWHAZ*. Expression was measured 30 min after the loading event. Analysis of variance was restricted to 3-weeks groups because only two donors were available at 6 weeks. *: *p* < 0.05, **: *p* < 0.01, ***: *p* < 0.001.

The stress response to DC from 30–40% strain was about 5× greater than that from 10–20% (Figure 6A). Although the initial dynamic stress responses for longer pre-culture periods were not always larger, the equilibrium dynamic stress responses for 6-weeks of pre-culture were at least double those for the 3-weeks groups (Figure 6A,B). There was much more matrix staining positively for sulphated proteoglycans in 6-weeks compared to 3-weeks groups for all three donors (Figure 6C). There was a general dose response for strain and the magnitude of *c-FOS*, *SO×9*, and *PTGS2* induction regardless of pre-culture duration, which was consistent with the much greater stress response under DC with higher strains. In contrast, the effects of longer pre-culture durations did not show a consistent trend for the two available donors: donor D had consistently higher fold inductions of measured genes for 3-weeks groups, whereas donor F outcomes were mixed (Figure 6D).

### Experiment III: Long-Term Mechano-Hypoxia Conditioning

**FIGURE 7:**
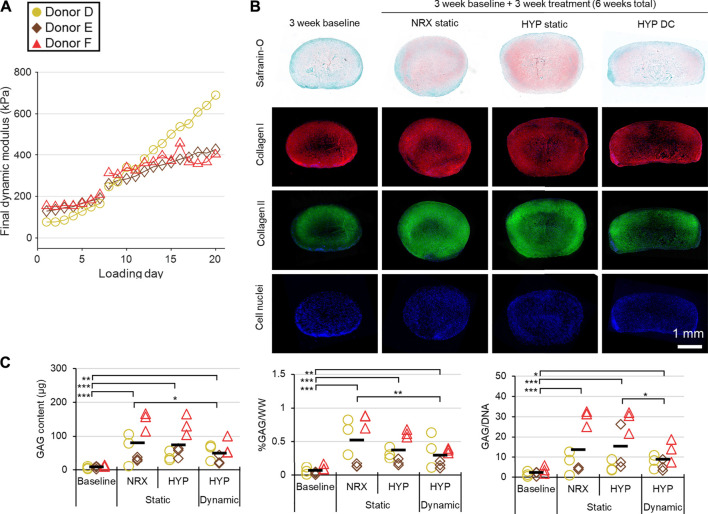
Experiment III: Mechanical and protein outcomes from 3 weeks of mechano-hypoxia conditioning after a 3-weeks baseline pre-culture period (up to a 6-weeks total culture duration). **(A)** Mechanical performance over time for each donor. The strain offset was increased from 30 to 35% on loading day 8, resulting in a jump in properties. For clarity, properties for only 1 of four loading events is presented per day. **(B)** Representative tissue matrix phenotypes. **(C)** Biochemistry. GAG, glycosaminoglycans; WW, wet weight. *: *p* < 0.05, **: *p* < 0.01, ***: *p* < 0.001.

**FIGURE 8:**
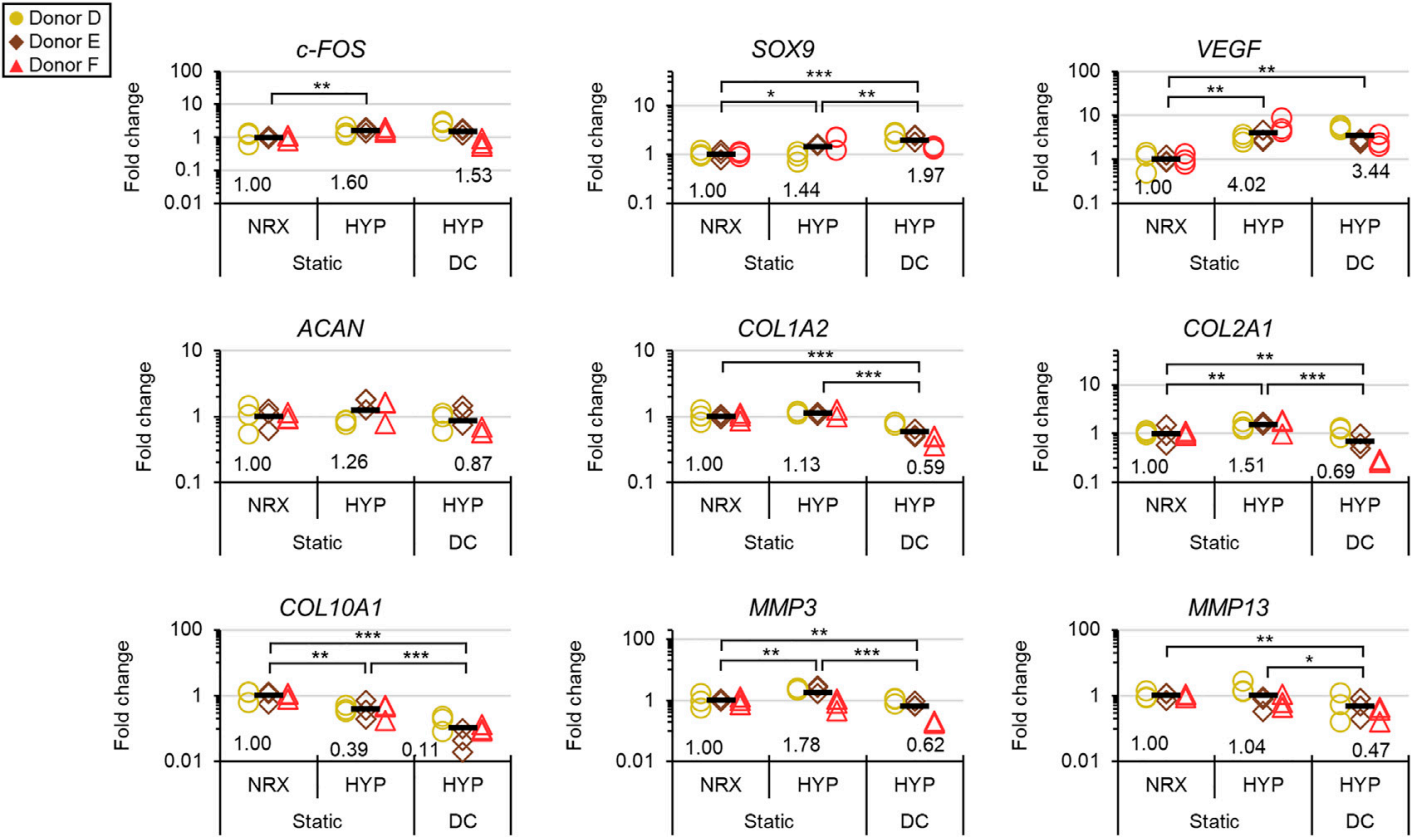
Experiment III: Gene expression by qRT-PCR after the 3-weeks mechano-hypoxia treatment. Expression was measured 6 h after the final loading event. Fold changes are with respect to the NRX/static group within each donor, and values for each gene of interest are normalized to the mean expression of housekeeping genes *β-actin*, *B2M*, and *YWHAZ*. *: *p* < 0.05, **: *p* < 0.01, ***: *p* < 0.001.

The dynamic modulus increased over the 3-weeks mechano-hypoxia conditioning period with some donors showing a sigmoidal growth curve (Figure 7A). The fold increase in equilibrium dynamic modulus during loading ranged from 1.8 to 5.1-fold after adjustment for the increased strain magnitude in weeks 2/3 (Figure 7A). Histologically, tissues in HYP/DC appeared more rectangular in cross-section due to the applied deformation and there were no differences among 6-weeks groups in collagen immunofluorescence (Figure 7B and Supplementary Figure S4.4B). A core region with reduced matrix staining and cell densities developed by the 3-weeks baseline and persisted in all groups for all donors (Figure 7B). There was also occasionally decreased cell density in these regions. Among 6-weeks groups, staining for hyaline cartilage-type matrix constituents was highest in HYP/stat and lowest HYP/DC, which was consistent with glycosaminoglycan (GAG) outcomes and mRNA expression of matrix precursors (Figures 7B,C, 8). *SOX9* expression was inconsistent with these outcomes, being highest in DC/HYP; this suggested that hyaline cartilage-related matrix gene transcription was being influenced by other factors (Figure 8). Hypertrophic markers were profoundly suppressed by mechano-hypoxia conditioning; for example, *COL10A1* was suppressed 2.5-fold by HYP alone and a further 3.5-fold by DC for a total of 9-fold suppression compared to NRX/stat (Figure 8).

## Discussion

This article investigated “mechano-hypoxia conditioning” as a strategy for tissue engineering the inner meniscus. Hypoxia (HYP) and dynamic compression (DC) 30–40% affected gene expression in human meniscus fibrochondrocyte (MFC)-based engineered tissues in an overwhelmingly additive manner after 5 days. It is likely that some more synergistic interactions existed than were detected but that detection power was limiting for the RNA-seq analysis. HYP and DC 30–40% also drove an increase in expression of hyaline cartilage-related precursors, indicating that the mechano-hypoxia treatment was promoting fibrocartilage formation. Thus, our hypothesis that the 5-days mechano-hypoxia treatment would have pro-fibrocartilage effects at the gene expression level was supported. Previously, Pauwels proposed that hydrostatic pressure, developing during compressive loading of tissues, is the specific stimulus to induce formation of cartilaginous matrix in the body (Pauwels, 1980). Krompecher later proposed that the hydrostatic pressure generated during mechanical loading forces vessels closed by overcoming blood pressure; this reduces blood supply to related tissues and results in a hypoxic environment that is instead the specific chondrogenic stimulus (Krompecher and Tóth, 1964). The outcomes of experiment I indicate that mechanical loading can be a direct promoter of cartilage-related gene expression independently of low oxygen. These effects were primarily driven by hypoxia rather than DC, however, which is generally consistent with Krompecher’s view. The previous studies that directly evaluate combined hypoxia and mechanical loading usually showed pro-chondrogenic effects of hypoxia but mixed outcomes from cyclic and intermittent hydrostatic pressure and dynamic compression (Domm et al., 2000; Hansen et al., 2001; Szojka et al., 2021a; Szojka et al., 2021b; Wernike et al., 2008; Meyer et al., 2010; Parker et al., 2013). One of these studies, applying porcine mesenchymal stem cells (MSCs), noted that, “low oxygen is a more potent promoter of chondrogenesis than dynamic compression.” The outcomes of experiment I (Figures 2, 4) affirm this statement for human MFCs at the gene expression level (Meyer et al., 2010).

Outcomes in experiments I/II suggest that the magnitude of the gene expression response to mechanical loading is closely related to the aggression of the loading regime (Figures 2, 6). To be useful for meniscus tissue engineering, Cyclic hydrostatic pressure (CHP) loading and hybrid displacement-load control regimes such as the DC 1%/2 kPa group introduced here will require more aggressive parameters than those used here, such as higher hydrostatic pressures (Gunja et al., 2009; Gunja and Athanasiou, 2010) and applied strains per cycle (e.g., 1.5%/2 kPa). Platen liftoff has long been recognized as a problem in DC loading studies and is typically addressed by use of static strain offsets or reduced loading frequencies (Mauck et al., 2000). The hybrid DC regime introduced here provides a unique solution to the problem of platen liftoff that could be useful in other tissue engineering applications of DC.

Experiment II showed that to induce gene expression changes: i. the early loading period is most important, and ii. there is a dose effect with increasing strain (Figures 5, 6). Previous researchers identified that DC systems reached a mechanical steady state within minutes, which we confirmed at the gene expression level (Puetzer and Bonassar, 2016). The (5c + 5r) × 6 group showed partial recovery of the stress response for each loading incident but without differential gene regulation to the other 60-min groups; longer cyclic rest periods may thus be more promising (Figure 5). The results complement what has been described as a fundamental rule in bone: the adaptive loading response saturates quickly, causing diminishing returns for extended loading incident durations (Turner, 1998). Many DC parameters need to be appropriately selected in tissue engineering to achieve beneficial outcomes (Anderson and Johnstone, 2017; Salinas et al., 2018). Loading events as short as 5 min make it much more feasible to optimize these parameters, which is a useful outcome for the tissue engineering field. The observed dose effect with strain (an increased static strain offset from 10 to 30% with a fixed dynamic strain amplitude of 10%) indicates that DC can directly induce *SOX9* expression rather than only doing so indirectly through enhanced medium mixing and platen-induced hypoxia (Figure 6). More investigation is required regarding the effects of pre-culture duration on gene regulation in response to DC, as the two available donors showed different trends.

Mechano-hypoxia conditioning (3 weeks) supported increases in mechanical properties with mixed matrix outcomes that included suppression of hypertrophy at the mRNA level (Figures 7, 8). Normal growth with culture time surely contributed to the mechanical gains as previously reported, but it is doubtful that it could have led to the sigmoid-shaped mechanical growth curves (Figure 7) (Szojka et al., 2021c). These could instead have been the product of factors such as inflammation early on and depletion of growth factors in the medium in the late stages. Each donor had a different growth profile, suggesting that efforts to tailor loading regimes on a donor-to-donor basis could be fruitful (Figure 7). The increase in final dynamic modulus in experiment II (for the available donors), conducted in parallel from 3 to 6 weeks in free-swelling culture compared to the mechano-hypoxia treatment in experiment III (after adjustment for higher strain levels in the second 2 weeks) were 2.2 vs. 5.1-fold (Donor D) and 1.3 vs. 1.8-fold (Donor F) (Figures 6, 7). This suggests that the mechano-hypoxia treatment indeed accelerated mechanical property development. Mechanical loading has previously been reported to have beneficial outcomes on mechanical properties (Baker et al., 2010; Huey and Athanasiou, 2011; Puetzer and Bonassar, 2016). DC led to superior outcomes at 2 but not 6 weeks compared to static controls with bovine MFCs (Ballyns and Bonassar, 2011), which may be consistent with the tendency for mechanical properties to plateau here. Matrix staining for sulphated proteoglycans appeared qualitatively reduced in HYP/DC compared to static controls, which could be due to GAG loss to the medium (Figure 7B) (Puetzer et al., 2012). There was no significant upregulation of *c-FOS* in HYP/DC, which is accounted for by the 6-h delay in harvest after the final loading event (Figure 8). Interestingly, matrix precursor expression in the mechano-hypoxia treatment was inferior to free-swelling controls despite HYP/DC having increased *SOX9*. Thus, altogether our hypothesis that extended mechano-hypoxia conditioning would lead to superior mechanical properties and extracellular matrix (ECM) protein expression was only partially supported. Future investigations are needed to understand the processes underlying this outcome in order to achieve gains in ECM properties with long-term loading (Figure 8) (Huang et al., 2010).

This study had several limitations. Inclusion of more donors in all experiments would have increased detection power; at best three donors were included in each experiment albeit with multiple tissue replicates and experimental designs favourable to detection of main effects, e.g., the factorial investigation in experiment I. A second limitation is that RNA-seq was only performed after the relatively short 5-days mechano-hypoxia treatment in experiment I. In longer-term mechano-hypoxia conditioning, tissue properties would diverge due to the unique gene expression profiles in each group (as identified in experiment I), likely affecting the reception of and response to mechano-hypoxia signals. Thus, many more gene interactions could be identified with longer-term treatments due to differing tissue properties; nevertheless, the results here indicate that the early molecular response to the treatments has few interactions (Figure 4A). The finding in experiment II that 5 min of loading was adequate to saturate much of the gene expression response was dependent on the state of maturity of the tissues (Figure 5). A longer course of loading than 5 min could be required for a more mature tissue that had undergone less stress relaxation by this time point; thus, to determine the appropriate loading incident duration for a different tissue the stress relaxation profile needs to first be assessed. Finally, experiment III had two main limitations. First, the medium was not changed during the whole 3-weeks culture period (though it was initially supplied in 5× excess and there was no colour change to indicate an accumulation of cell metabolites). It is possible that the growth factors degraded during this time, resulting in a tendency for mechanical properties to plateau (Figure 7). Second, the static control groups were free swelling due to the limited number of bioreactors available; it would have been ideal to have 0, 30, and 40% static compression controls within replicate bioreactors. These would have accounted for the reduced exposed surface area in HYP/DC, which likely contributed to the outcome of reduced matrix staining in HYP/DC compared to the free-swelling controls.

A priority in future work would be to address the matrix-poor core regions by increasing the local density of viable ECM-forming cells. Possibilities include using a gentle DC regime to promote media supply to the inner regions, forced perfusion during the pre-culture phase, or constructs incorporating medium channels (Timmins et al., 2007; Schulz et al., 2008; Daly et al., 2018). As well, the suppression of hypertrophic markers in combined HYP/DC suggests mechano-hypoxia conditioning may have utility in engineering inner meniscus and articular cartilage with MSCs. These have some superior functional matrix-forming capabilities to MFCs but with higher native expression of constituents such as type X collagen that are undesirable for engineering cartilaginous tissues (Elkhenany et al., 2020). The bioreactor in experiment III required little user input during the culture period, which is practical for long-term investigations of mechanical loading that are usually labour intensive. Mechano-hypoxia conditioning with a similar bioreactor could also be employed towards building a functional meniscus. The timing of the treatment could be delayed until an organized and appropriately mature matrix of collagen fibres was formed within a biomimetic meniscus shape, potentially using boundary conditions and cell contraction or polymer fibres with a compression or tension-loading component to guide fibre formation (Ballyns and Bonassar, 2011; Puetzer and Bonassar, 2016; Puetzer et al., 2021). Then, mechano-hypoxia conditioning could be initiated to promote mechanical property development and fibrocartilage maturation in the inner regions, mimicking an aspect of human meniscus development.

In conclusion, mechano-hypoxia conditioning is promising for meniscus tissue engineering as it may support fibrocartilage formation and compressive property development. However, further research is required to achieve continual development of mechanical properties towards native levels and to incorporating a native meniscus-like collagen architecture.

## Data Availability

The RNA-seq dataset can be found in the online repository below: https://www.ncbi.nlm.nih.gov/geo/query/acc.cgi?acc=GSE180467. The remaining quantitative data is available in the Supplementary Materials section.
