# Supplementary material for: Mechano-Hypoxia Conditioning of Engineered Human Meniscus
Source: Front Bioeng Biotechnol. 2021 Sep 3;9:739438. doi: 10.3389/fbioe.2021.739438 (PMC8446439; doi:10.3389/fbioe.2021.739438)
Supplement: Supplementary file 1 [file Table1.docx]

Supplementary Tables

**Supplementary Table 1.** Meniscus tissue donor details. Shape and colour refer to how donors are presented in quantitative data figures.

| Donor # | Sex/Age | Population doublings | Shape | Colour |
| --- | --- | --- | --- | --- |
| A | Male/21y | 6.3 | Circle | Blue |
| B | Male/22y | 7.7 | Diamond | Green |
| C | Female/45y | 6.0 | Triangle | Orange |
| D | Female/21y | 7.2 | Circle | Mustard |
| E | Female/25y | 5.3 | Diamond | Brown |
| F | Male/40y | 6.5 | Triangle | Red |

**Supplementary Table 2.** Primers sequences for qRT-PCR.

| Gene | Forward | Reverse | GenBank |
| --- | --- | --- | --- |
| *ACAN* | AGGGCGAGTGGAATGATGTT | GGTGGCTGTGCCCTTTTTAC | NM_001135.3 |
| *ADAMTS1* | GCTCATCTGCCAAGCCAAAG | ATCTACAACCTTGGGCTGCAA | NM_006988.5 |
| *β-actin* | AAGCCACCCCACTTCTCTCTAA | AATGCTATCACCTCCCCTGTGT | NM_001101.4 |
| *B2M* | TGCTGTCTCCATGTTTGATGTATCT | TCTCTGCTCCCCACCTCTAAGT | NM_004048.3 |
| *c-FOS* | CGTCTCCAGTGCCAACTTCA | GGTCCGGACTGGTCGAGAT | NM_005252.4 |
| *CCN1* | AAATCCCCCGAACCAGTCA | GGGCCGGTATTTCTTCACACT | NM_001554.5 |
| *COL1A1* | CAGCCGCTTCACCTACAGC | TTTTGTATTCAATCACTGTCTTGCC | NM_000088.4 |
| *COL1A2* | GCTACCCAACTTGCCTTCATG | GCAGTGGTAGGTGATGTTCTGAGA | NM_000089.3 |
| *COL10A1* | GAAGTTATAATTTACACTGAGGGTTTCAAA | GAGGCACAGCTTAAAAGTTTTAAACA | NM_000493.3 |
| *COL2A1* | CTGCAAAATAAAATCTCGGTGTTCT | GGGCATTTGACTCACACCAGT | NM_001844.5 |
| *MMP1* | ATGAGTCTTTGCCGGAGGAA | GTGACACCAGTGACTGCACATG | NM_002421.4 |
| *MMP13* | CATCCAAAAACGCCAGACAA | CGGAGACTGGTAATGGCATCA | NM_002427.4 |
| *MMP3* | AGGCATCCACACCCTAGGTTT | ATCAGAAATGGCTGCATCGAT | NM_002422 |
| *NR4A1* | AGCATTATGGTGTCCGCACAT | TGCACTGTGCGCTTGAAGA | NM_001202234.2 |
| *PTGS2* | GAATCATTCACCAGGCAAATTG | TGCGGGTGGAACATTCCTA | NM_000963.4 |
| *SOX9* | CTTTGGTTTGTGTTCGTGTTTTG | AGAGAAAGAAAAAGGGAAAGGTAAGTTT | NM_000346.3 |
| *VEGF* | GCACGGTCCCTCTTGGAA | CGGTGATTTAGCAGCAAGAAAA | NM_001025366.3 |
| *YWHAZ* | TCTGTCTTGTCACCAACCATTCTT | TCATGCGGCCTTTTTCCA | NM_003406.3 |
