# Supplementary material for: Mechano-Hypoxia Conditioning of Engineered Human Meniscus
Source: Front Bioeng Biotechnol. 2021 Sep 3;9:739438. doi: 10.3389/fbioe.2021.739438 (PMC8446439; doi:10.3389/fbioe.2021.739438)
Supplement: Supplementary file 2 [file DataSheet1.docx]

Supplementary Document

# This document has four sections:

1. Seeding Density Pilot Study
2. Orbital Shaking Pilot Study
3. Loading Days Comparison
4. Miscellaneous Supplementary Figures

# Seeding Density Pilot Study

This pilot study aimed to determine whether to include a high seeding density in the main experiments of hypoxia and mechanical loading. We performed a basic monolayer assessment and then compared densities of 5 and 30×10^6^ cells/cm^3^ with a control group without adjustment of the supplied medium volume for one donor. The high seeding density was not clearly advantageous to the standard one and was thus not used in the main experiments.


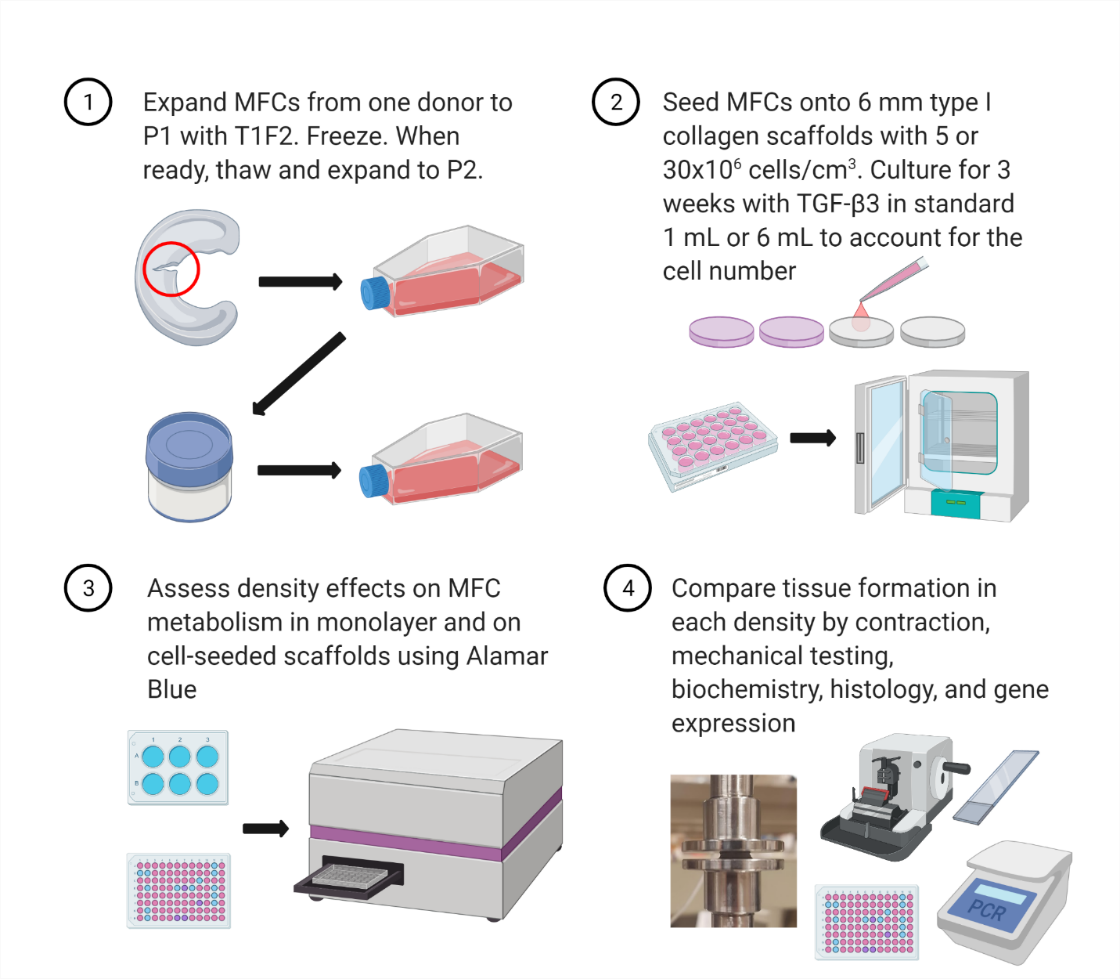


**Supplementary Document Figure 1.1.** Seeding density experiment overview. The medium for expansion and tissue culture was the same as in the main experiments. MFCs: Meniscus fibrochondrocytes. T1F2: TGF-β1 and FGF-2. The donor was the same as Donor 3 used in experiment III. Created using Biorender.com (2021).


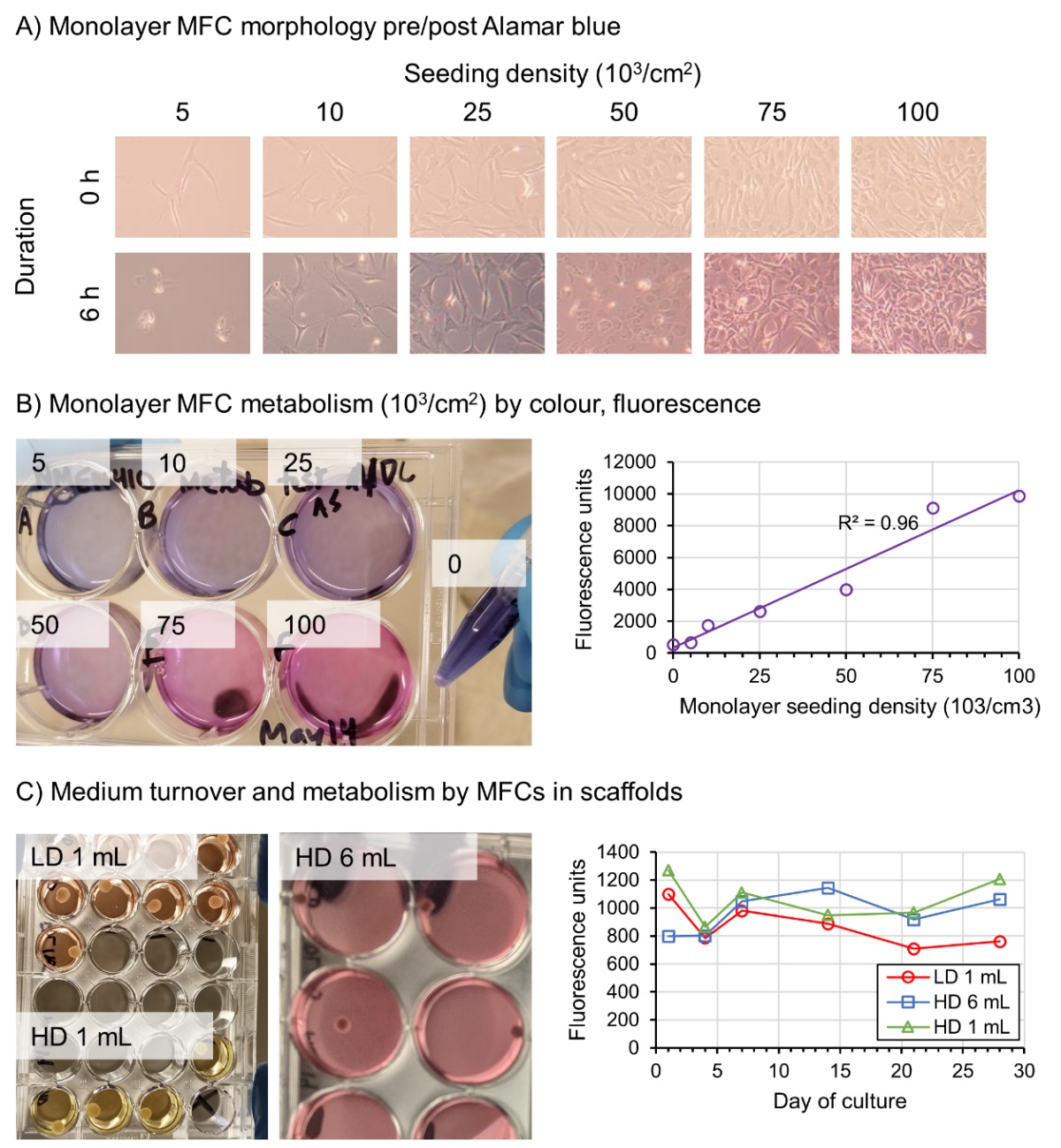


**Supplementary Document Figure 1.2.** (A) 24h after seeding cells into 6 well plates at the indicated densities in expansion medium (but without T1F2), the cells were treated with 90% expansion medium (without T1F2) + 10% Alamar blue reagent for 6h (Thermofisher, USA). The Alamar blue treatment seemed to cause adverse changes in cell morphology in monolayer. (B) Colour change after the 6h treatment and fluorescence measurement. (C) Left: Tissue culture medium turnover after 3 days. Right: Alamar blue turnover after 30-minute treatments in 90% tissue culture medium repeated every few days on the same replicate construct. Alamar blue turnover did not show a clear relationship to the number of seeded cells in 3D. It seemed to instead be related to the surface area of the constructs. LD: low density (5×10^6^/cm^3^), HD: high density (30×10^6^/cm^3^).


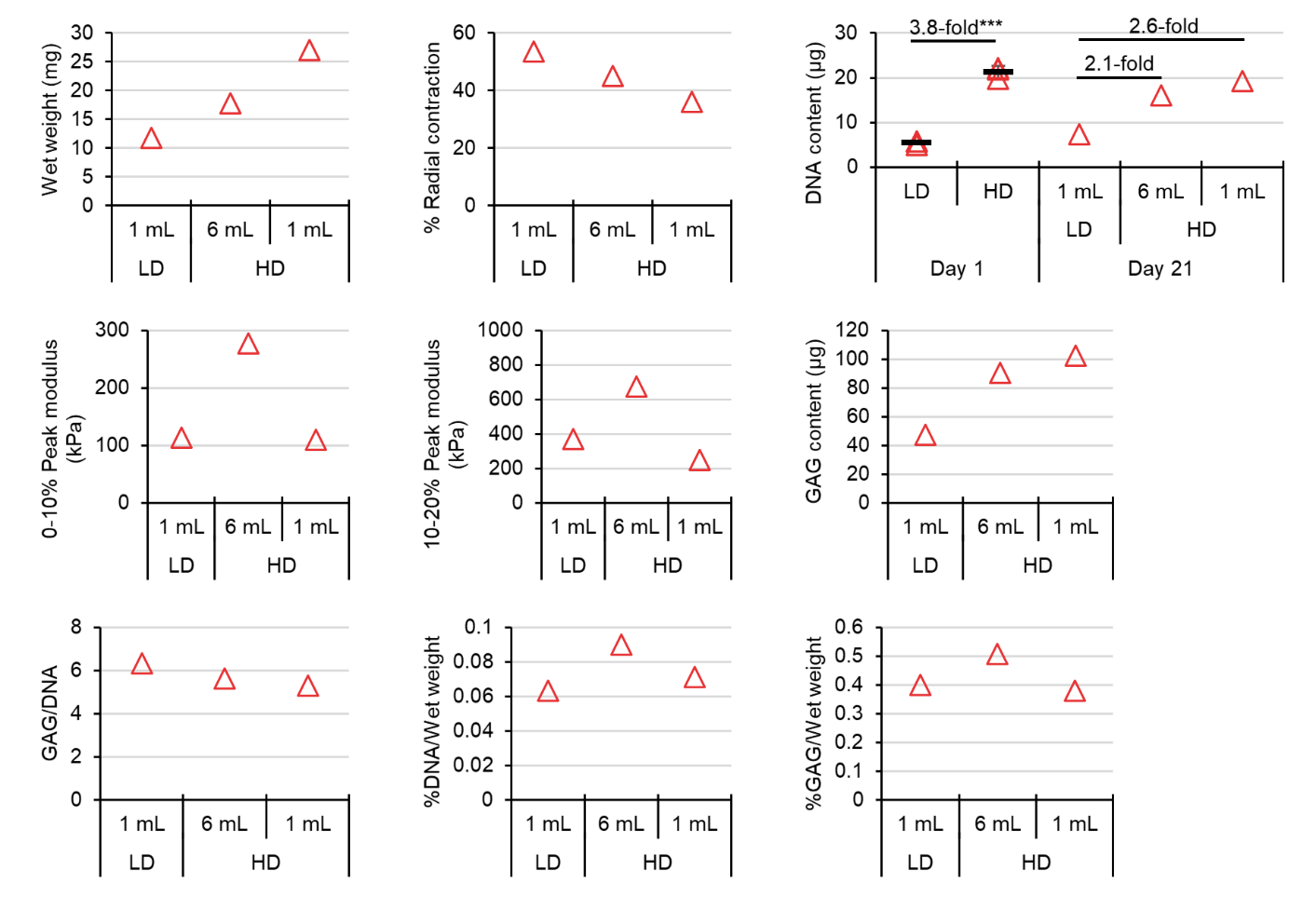


**Supplementary Document Figure 1.3.** Contraction, mechanical, and biochemical analysis after 3 weeks of tissue culture. Samples were tested in stress relaxation tests at a strain rate of 50% strain/s. DNA contents did not show a 6-fold difference on day 1 as had been expected. When seeding scaffolds, a cell pellet containing sufficient cells to seed all replicate scaffolds at the appropriate density is suspended in 25 μL of medium per scaffold. Each scaffold is then seeded with 25 μL of the suspension. The lower-than-expected DNA on day 1 in HD relative to LD reflects error in the assumption of negligible cell volumes during the cell seeding protocol, because the volume of cells is less trivial at higher densities relative to the volume of medium to seed each scaffold (25 μL) (Donor F).


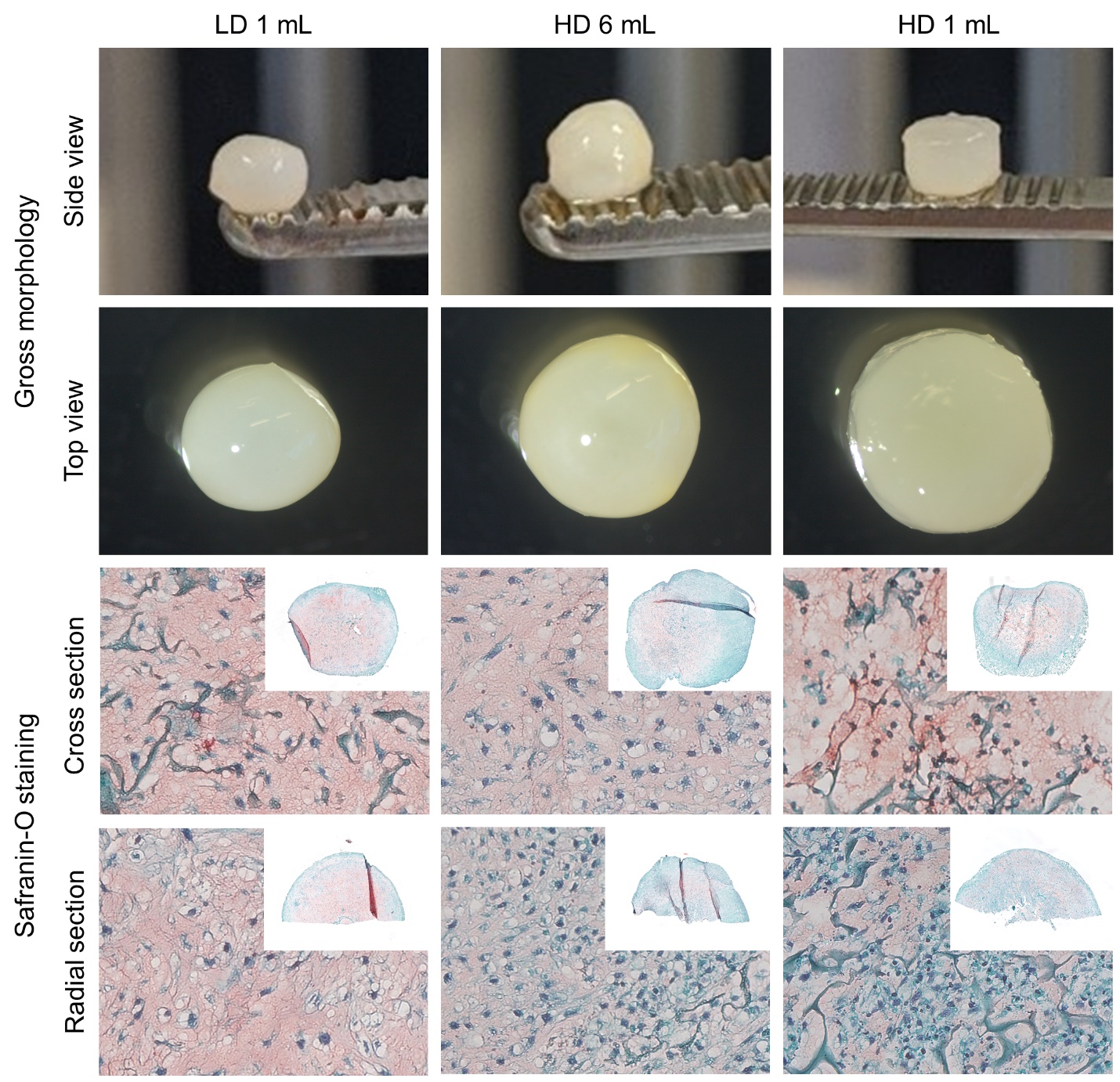


**Supplementary Document Figure 1.4.** Morphology and Safranin-O/Fast Green FCF/Haematoxylin staining.


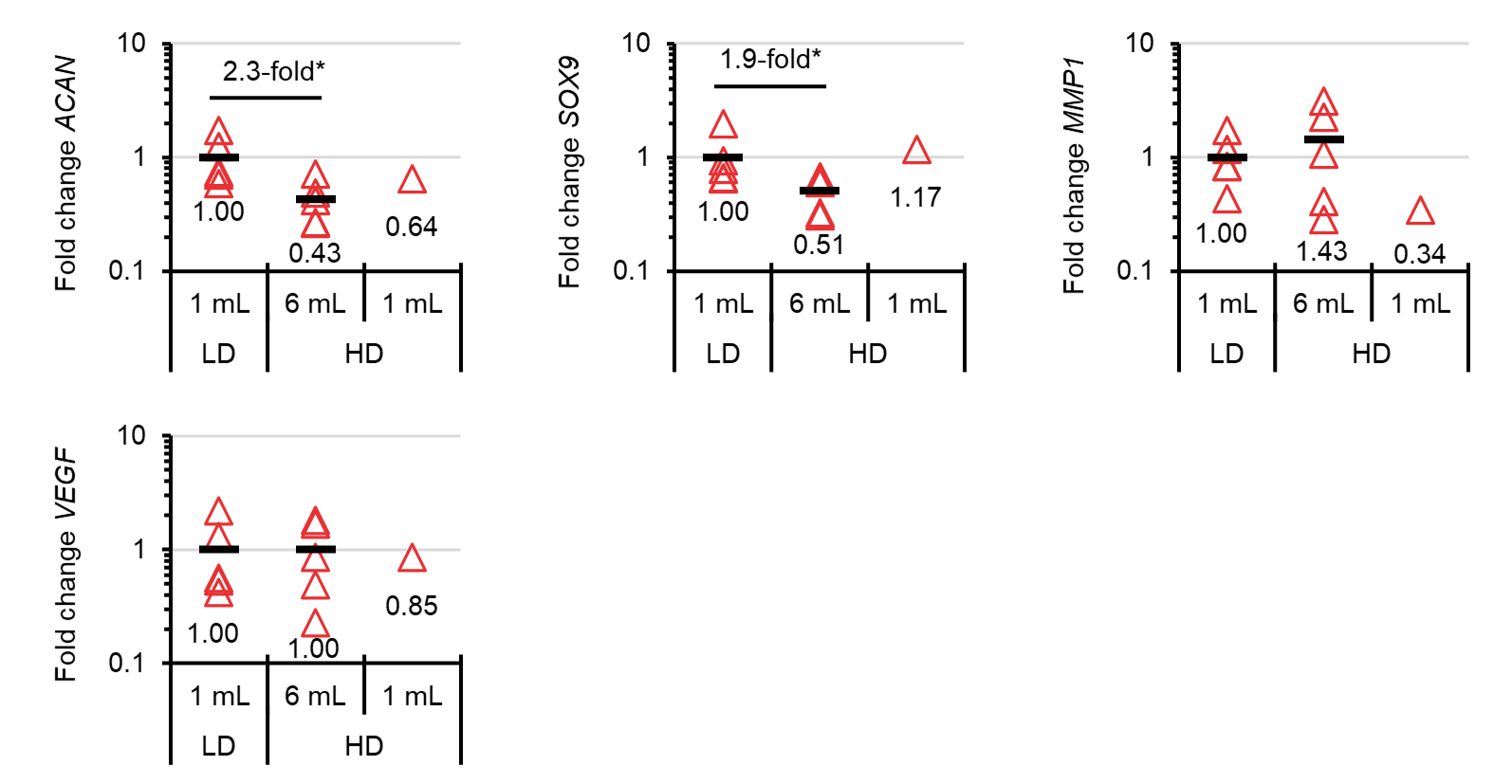


**Supplementary Document Figure 1.5.** mRNA expression levels measured by qRT-PCR compared to LD 1 mL in each group. Only one replicate was seeded in the HD 1 mL group for gene expression.

# Orbital Shaking Pilot Study

This pilot study aimed to determine whether pre-culture on an orbital shaker/rotator was useful for the main experiments, e.g., by improving matrix synthesis through increased nutrient transfer. The benefits of shaking were not obvious for the one donor tested. Shaking caused increased scaffold contraction, which was undesirable because it promoted deviation from a cylindrical tissue shape. Thus, shaking was not used beyond experiment I.


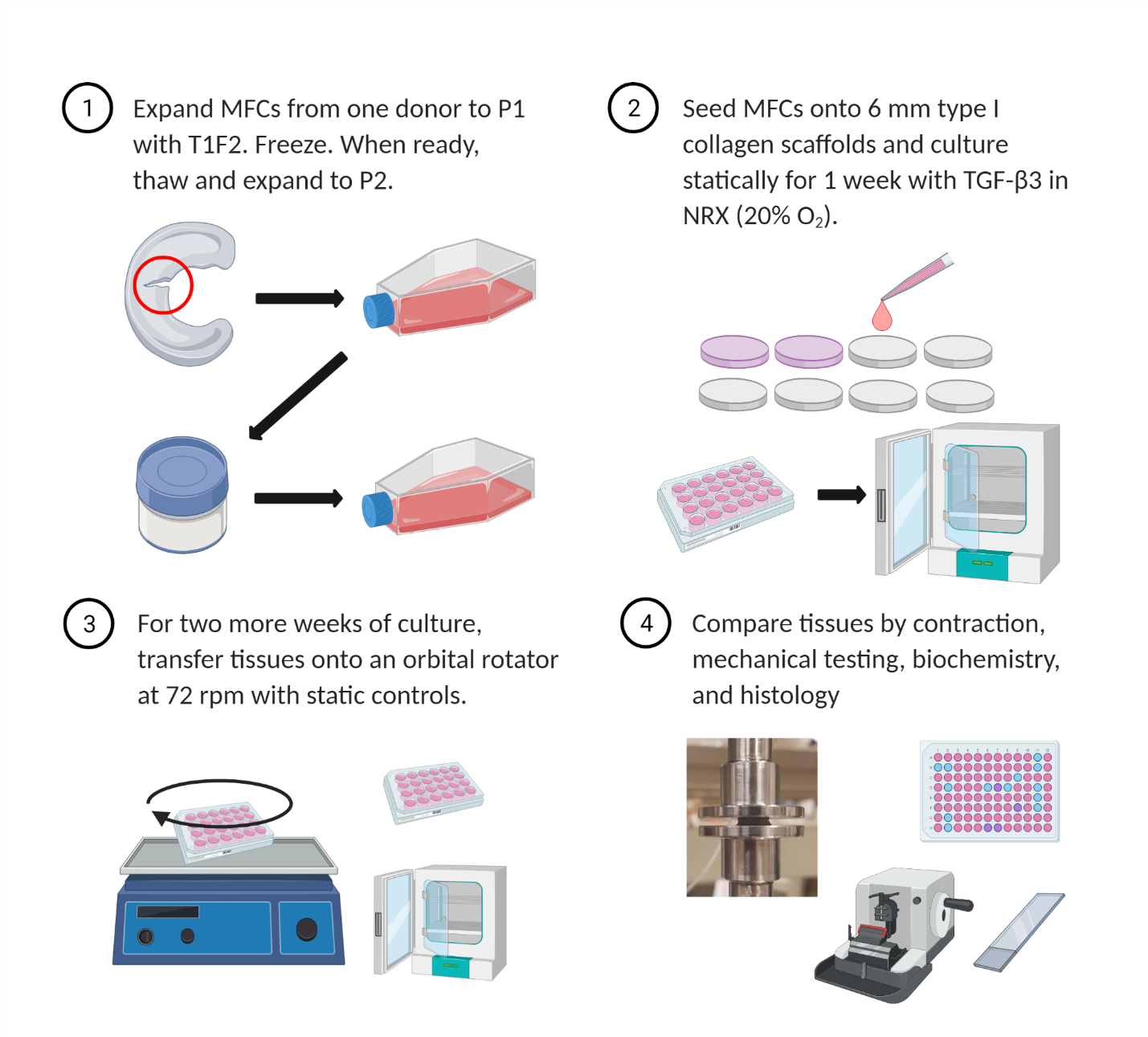


**Supplementary Document Figure 2.1.** Orbital shaking pilot experiment. The donor was the same as Donor 1 used in experiments I & II. Created using Biorender.com (2021).


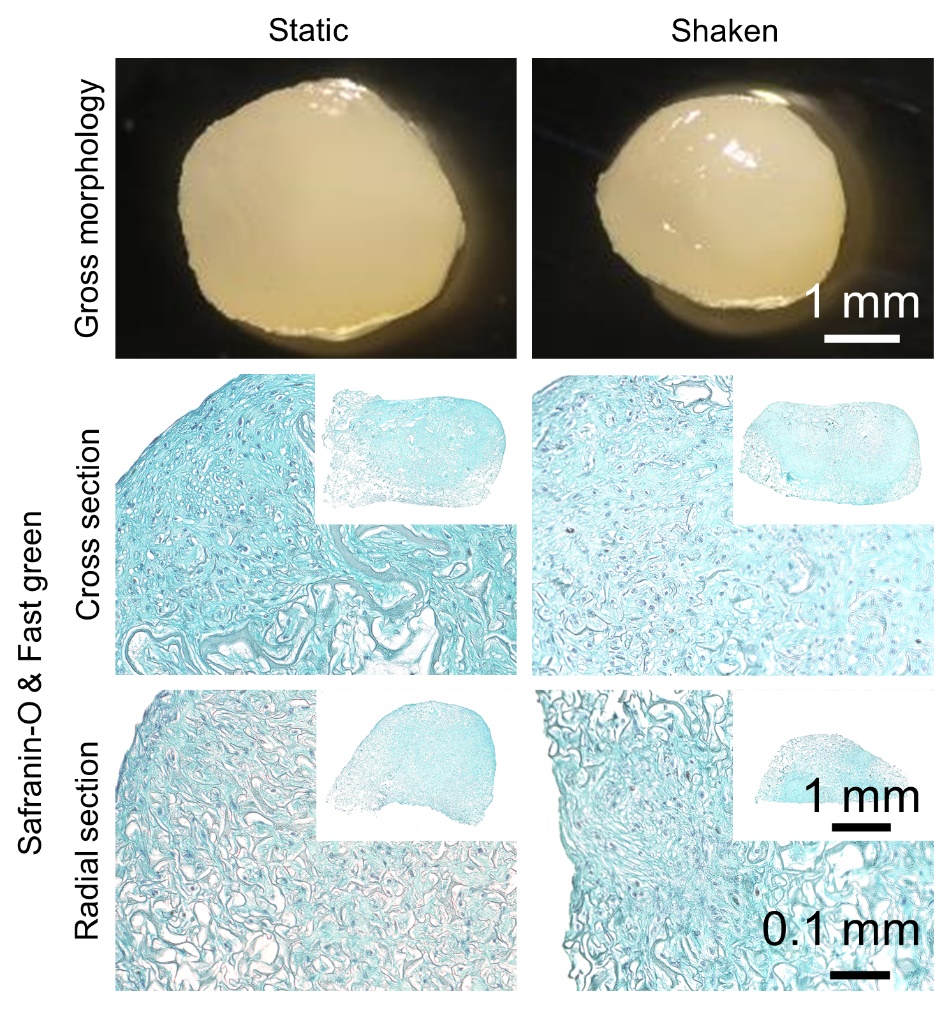


**Supplementary Document Figure 2.2.** Gross morphology and Safranin-O staining of static vs shaken tissues.


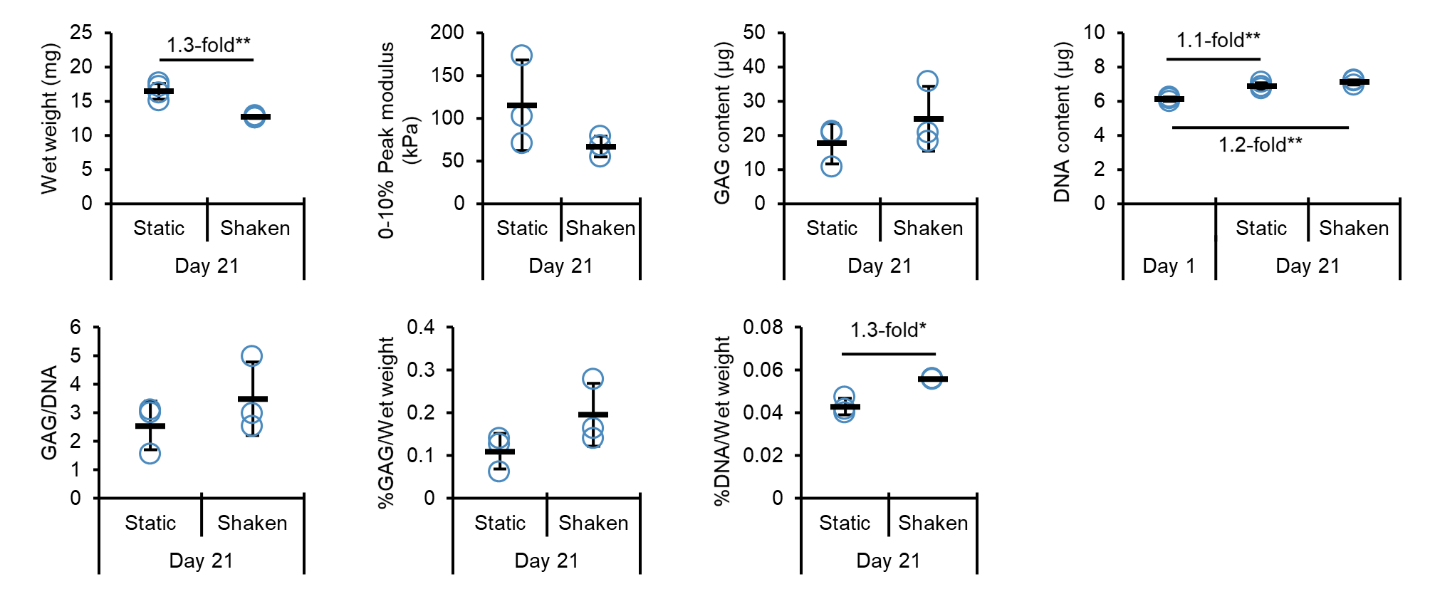


**Supplementary Document Figure 2.3.** Wet weight and mechanical and biochemical analysis of static vs shaken tissues (Donor A).

# Loading Days Comparison

For the three donors in experiment I, we included control groups that were loaded on day 1 using the DC 1%/2 kPa group and harvested. *c-FOS* induction was higher on the first day of loading and *ACAN* and *MMP1* expression were higher on day 5 regardless of loading, which likely reflects the increased culture time with TGF-β3 supplementation.


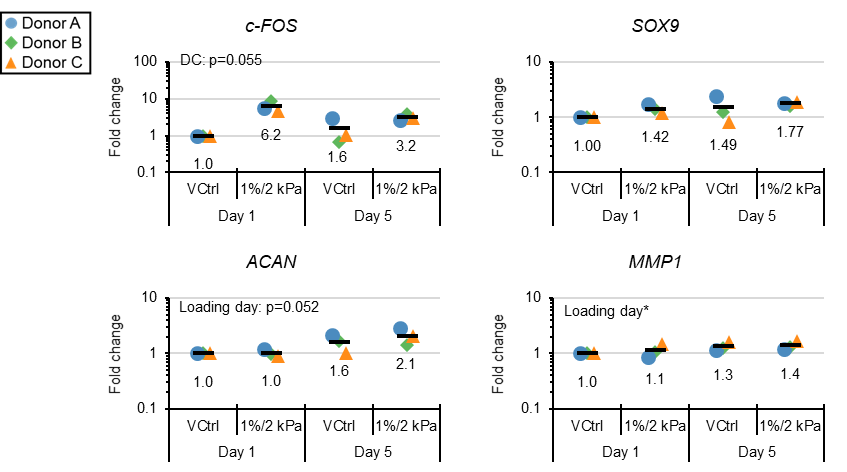


**Supplementary Document Figure 3.1.** Experiment I: mRNA expression levels measured by qRT-PCR compared to Day 1 VCtrl in each group. Tissues were loaded either 1 or 5 days in NRX for 1h with the 1%/2 kPa regime.

# Miscellaneous Supplementary Document Figures


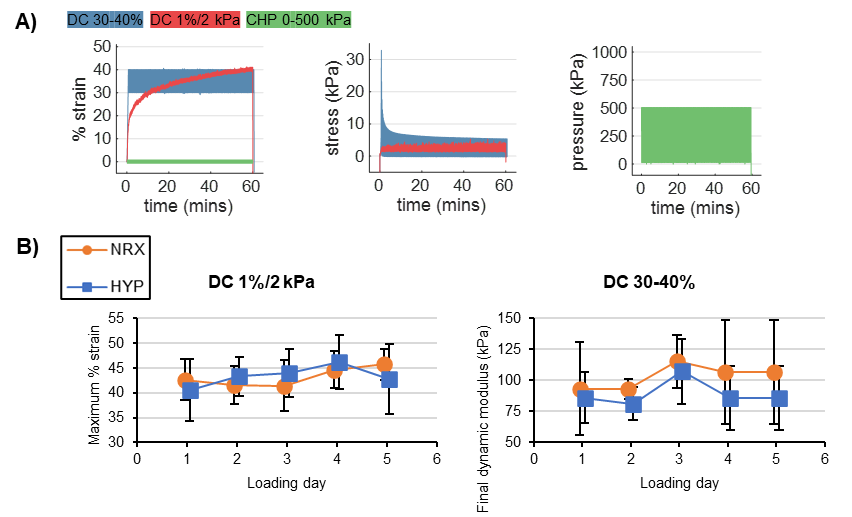


**Supplementary Document Figure 4.1.** (A) Representative loading curves in each mechanical loading group. (B) Measures of mechanical competency in DC groups over time by oxygen tension.


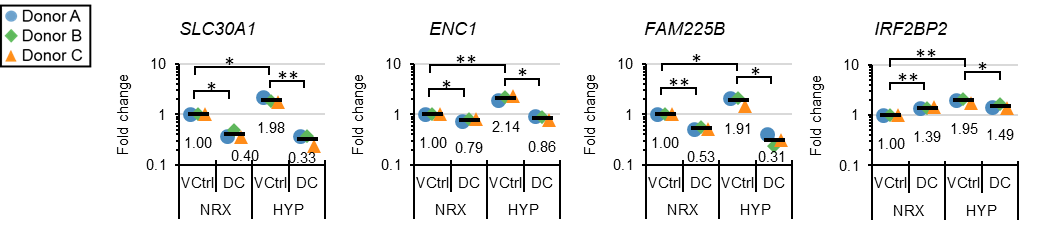


**Supplementary Document Figure 4.2.** Experiment I: Genes having significant (q<0.05) interactions and total count≥250. Groups were compared by ANOVA with mechanical loading and oxygen tension as fixed factors and donor as a random factor. *: p<0.05, **: p<0.01, ***: p<0.001.


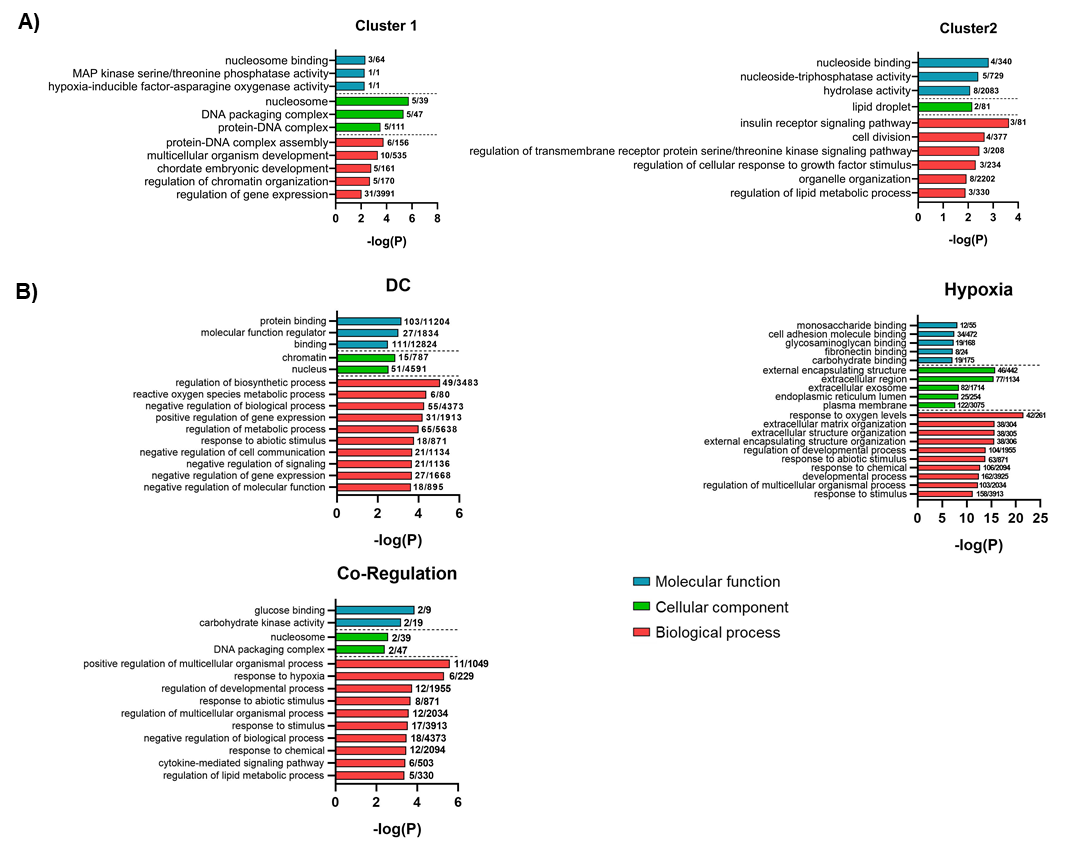


**Supplementary Document Figure 4.3.** Gene ontology (GO). The fractions are the number of genes matching the GO term in the list divided by the total number of genes described by the GO term. (A) GO for the top two interaction clusters (p<0.01). (B) GO for the individual treatments and co-regulation (q<0.05 and total count>250).


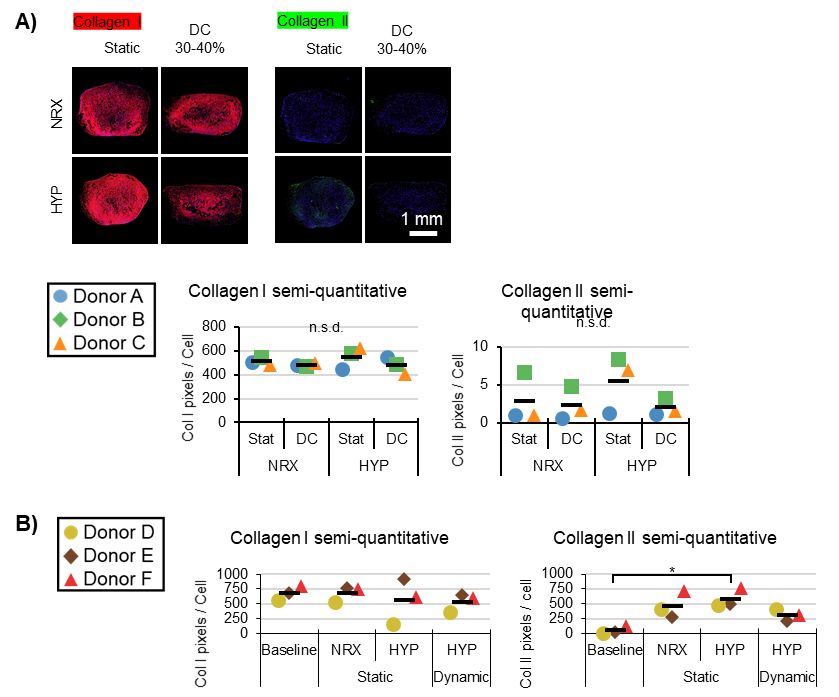


**Supplementary Document Figure 4.4.** Immunofluorescence and semi-quantitative analysis for types I and II collagens. (A) Experiment I: 5-day mechano-hypoxia treatment. (B) Experiment III: 3-week mechano-hypoxia treatment. N.s.d.: no significant differences. ANOVA was performed with donor as a random factor. *: p<0.05.


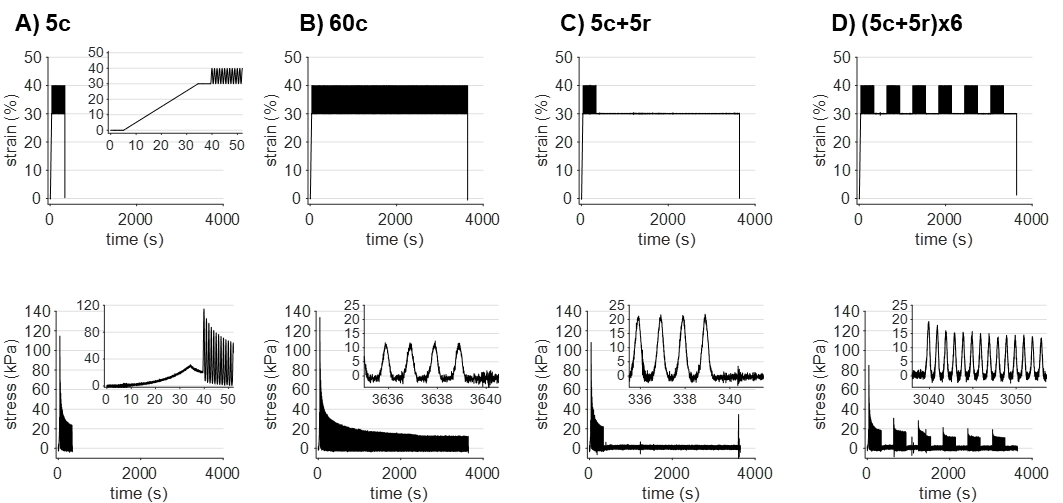


**Supplementary Document Figure 4.5.** Experiment II: (A-D) Representative strain and stress vs. time for the four loading groups. The magnified subpanels show regions of interest for each curve.


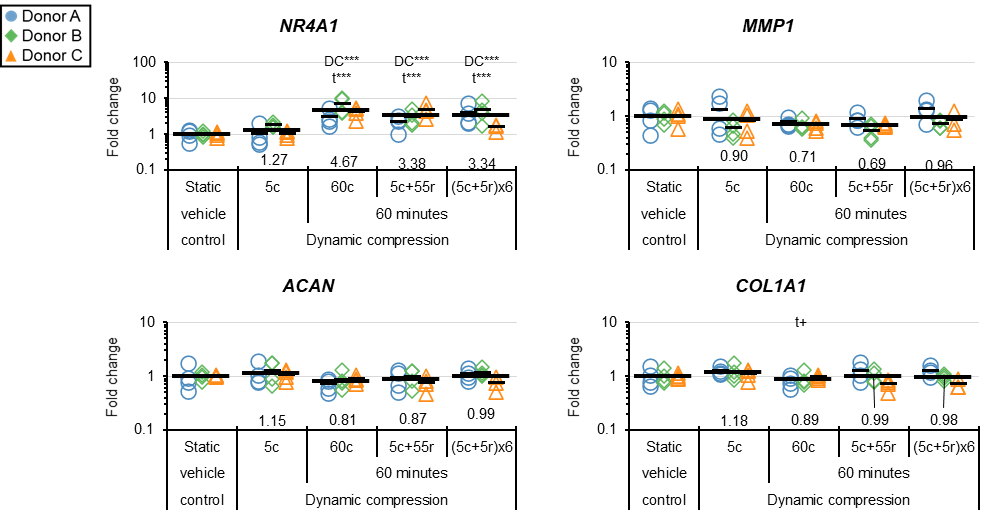


**Supplementary Document Figure 4.6.** Experiment II: Expression of additional genes. DC indicates that a group is significantly different from the static vehicle control. “t” indicates that a DC group is significantly different than 5c. +: p<0.10, ***: p<0.001.
